# A Comparison of Response Patterns for Progression-Free Survival and Overall Survival Following Treatment for Cancer With PD-1 Inhibitors: A Meta-analysis of Correlation and Differences in Effect Sizes

**DOI:** 10.1001/jamanetworkopen.2018.0416

**Published:** 2018-06-22

**Authors:** Bishal Gyawali, Spencer Phillips Hey, Aaron S. Kesselheim

**Affiliations:** 1 Department of Clinical Oncology and Chemotherapy, Nagoya University Hospital, Nagoya, Japan; 2 Program on Regulation, Therapeutics, and Law (PORTAL), Division of Pharmacoepidemiology and Pharmacoeconomics, Brigham and Women’s Hospital, Boston, Massachusetts; 3 Harvard Center for Bioethics, Harvard Medical School, Boston, Massachusetts

## Abstract

**Importance:**

Based on efficacy results from pivotal randomized clinical trials, PD-1 (programmed cell death 1) inhibitors, such as nivolumab and pembrolizumab, have been approved to treat various cancers. Response patterns with varying effects on progression-free survival (PFS) and overall survival (OS) have been reported for these drugs.

**Objective:**

To compare 2 outcomes for PD-1 inhibitors: the correlation between PFS and OS and the differences in treatment effect size between PFS and OS.

**Data Sources:**

A systematic search of PubMed, Google Scholar, the Cochrane Library, Web of Science, and conference abstracts for randomized clinical trials of nivolumab and pembrolizumab published in English.

**Study Selection:**

Randomized clinical trials of nivolumab or pembrolizumab in adults with solid-tissue cancers with a nonimmunotherapy control.

**Data Extraction and Synthesis:**

Two reviewers screened the studies for selection and extracted data on medians and hazard ratios (HRs) for PFS and OS. A pooled meta-analysis was conducted.

**Main Outcomes and Measures:**

Across all trials, correlation coefficients between median PFS and median OS and between PFS benefit and OS benefit as well as the HRs of PFS and OS were assessed. The difference in treatment effect sizes between PFS and OS was assessed using a ratio of HRs (rHR). Subgroup analyses were conducted to observe differences based on drug, tumor type, and timing of therapy.

**Results:**

Ten randomized clinical trials that included 4653 patients and met inclusion criteria were identified, as were 2 others (comprising 764 patients) in which nivolumab or pembrolizumab was used following treatment with ipilimumab. The correlations between median PFS and median OS (*r* = 0.676; *R^2^* = 0.457; *P* = .09) and the correlations between the change in PFS and the change in OS (*r* = 0.474; *R^2^* = 0.225; *P* = .28) were not significant. However, the correlation between HRs of PFS and OS was significant (*r* = 0.637; *R^2^* = 0.406; *P* = .048). Using random-effects meta-analysis, the protective effects of treatment were greater for OS than for PFS (pooled rHR, 1.18; 95% CI, 1.06-1.31; *P* = .002). There was no statistical evidence for heterogeneity across the studies (*Q* = 6.24; *P* = .72; *I^2^* = 0%). Subgroup analyses showed some differences in the treatment effect sizes based on drug type, tumor type, and line of therapy.

**Conclusions and Relevance:**

There was no significant correlation between OS and PFS in terms of medians and gains in medians, but their HRs were significantly correlated. The protective effects of treatment were greater for OS than for PFS. Traditional Response Evaluation Criteria in Solid Tumors–based PFS cannot capture the benefit of PD-1 inhibitors in patients with solid tumors, and OS should remain the gold standard.

## Key Points

**Question:**

Does the treatment effect size differ between overall survival and progression-free survival for PD-1 (programmed cell death 1) inhibitors used in patients with advanced solid tumors, and are overall survival and progression-free survival correlated?

**Findings:**

This meta-analysis of 12 randomized clinical trials found no significant correlation between overall survival and progression-free survival in terms of medians and gains in medians, but their hazard ratios were significantly correlated. The protective effects of treatment were greater for overall survival than for progression-free survival.

**Meaning:**

Progression-free survival cannot adequately capture the benefit of PD-1 inhibitors; thus, overall survival should remain the gold standard end point for trials of PD-1 inhibitors.

## Teaser

This meta-analysis of randomized trials of nivolumab and pembrolizumab for treatment of cancer (lung, renal, head and neck) and melanoma estimates correlations and effect size differences between the trials’ outcome measures of progression-free and overall survival.

## Introduction

The most important clinical outcome that can be observed among new cancer drugs is an improvement in overall survival (OS) when compared with current therapies. However, improvements in OS can take time to recognize and can be contaminated by crossover or the effects of postprogression therapies. As a result, progression-free survival (PFS) is often used as a surrogate for OS. But PFS has also been criticized as unreliable in some circumstances, as *progression* is defined as an increase in tumor size beyond an arbitrary cutoff and is prone to bias, particularly when the investigators are not blinded.^1^

When PFS strongly correlates with OS, PFS can be a useful and valid surrogate measure for evaluating a new therapy’s clinical effectiveness. However, this correlation has been shown to vary across treatment settings. For example, a 2015 systematic review showed that for most tumor types, there was only a weak correlation between anticancer drug–related changes in PFS and OS.^2^ A recent meta-analysis of targeted anticancer therapies showed that the drugs had a greater effect on PFS than OS, but that PFS benefits often did not translate to OS benefits.^3^

Two PD-1 (programmed cell death 1) inhibitor antibodies, nivolumab (Opdivo) and pembrolizumab (Keytruda), have been approved by the US Food and Drug Administration (FDA) based on efficacy in treating certain types of solid tumors, including advanced melanoma, lung cancer, renal cell cancer, urothelial cancer, Hodgkin-type lymphoma, head and neck cancer, and hepatocellular cancer. These PD-1 inhibitors show unconventional patterns of response, including long duration of responses, responses after initial progression (pseudoprogression), and even responses after discontinuation of therapy.^4,5^ This atypical response pattern is also observed in the crossing over of PFS curves in some randomized clinical trials (RCTs) of PD-1 inhibitors.^6^

The correlation between PFS and OS has not yet been formally studied across PD-1 inhibitors. To help guide future PD-1 research, we conducted a correlation and meta-analytic study of RCTs with PD-1 inhibitors in adult patients with solid tumors and evaluated the differences in treatment effect sizes between PFS and OS.

## Methods

### Study Identification

We conducted a systematic search of PubMed, the Cochrane Library, Web of Science, and Google Scholar for all RCTs of nivolumab and pembrolizumab in accordance with the Preferred Reporting Items for Systematic Reviews and Meta-analyses (PRISMA) reporting guideline. We used the search terms “nivolumab” or “opdivo” or “pembrolizumab” or “keytruda” or “pd-1” and limited our search to RCTs and findings published in English. The first search was performed on May 8, 2017, and reinforced on July 4, 2017. Relevant conference abstracts were also searched for updated data. After title and abstract screening by 2 independent investigators, the full texts of potentially relevant studies were downloaded and reviewed for the following exclusion criteria: (1) not an RCT, (2) not reporting data for PFS and OS, and (3) not reporting original data. Because the aim of our study was to evaluate the correlation between PFS and OS and the difference in treatment effect sizes between PFS and OS in solid tumors in adults, we also excluded studies involving pediatric patients, patients with hematological malignancies, RCTs comparing combinations of immunotherapies (eg, nivolumab plus ipilimumab), and RCTs that involved immunotherapy control groups (eg, ipilimumab control) (eFigure 1 in the Supplement).

Although we retained RCTs evaluating nivolumab and pembrolizumab as any line of therapy, for our primary analysis we excluded trials that tested these PD-1 inhibitors in populations that had already received another checkpoint inhibitor, such as CTLA-4 (cytotoxic T-lymphocyte–associated protein 4) or PD-L1 (programmed cell death 1 ligand 1) inhibitors, because the effect on PFS or OS could be a residual effect from the previous therapy. These studies were included as a secondary analysis.

### Data Extraction and Methodological Quality Assessment

This study was not submitted for institutional review board approval because it did not involve individual patient information and all data extractions were made from publicly available published articles. Data were independently extracted from published reports by 2 of us (B.G. and S.P.H.), with any discrepancy resolved through consensus of all authors. We collected key trial characteristics: treatment setting, primary end point, sample size, and details of the treatment and control regimens. For trial outcomes, we extracted the median PFS, median OS, and hazard ratios (HRs) with confidence intervals for PFS and OS.

We graded the quality of each trial using the 5-point Jadad scale for rating RCTs,^7^ with scores of 4 and 5 determined a priori to represent high-quality trials.

### Statistical Analysis

The primary end point of this study was the correlation and difference in treatment effect sizes between PFS and OS. We defined PFS as the time from randomization to first documented tumor progression or death from any cause and OS as the time from randomization to the date of death due to any cause. We used the Response Evaluation Criteria in Solid Tumors (RECIST), in which progression is defined as at least a 20% increase in the sum of diameters of target lesions, taking as reference the smallest sum on study plus an absolute increase of at least 5 mm or the appearance of any new lesions.

The correlation between median PFS and OS was assessed using the Pearson correlation coefficient. The PFS benefit was defined as median PFS of the PD-1 inhibitor group minus that of the control group, while OS benefit was defined as median OS of the PD-1 inhibitor group minus that of the control group. The correlation between the PFS benefit and the OS benefit and that between HR of PFS and HR of OS were also assessed using the Pearson correlation coefficient. All correlation studies were performed using SPSS statistical software version 22.0 (IBM) and Stata statistical software version 15 (StataCorp), and pooled meta-analyses were performed using R statistical software version 3.2.3 (R Project for Statistical Computing). We also conducted linear regression analysis to quantify the benefit in OS for a given magnitude of benefit in PFS whenever the correlation was significant.

To study the difference in treatment effect sizes between the PFS and OS, we used the ratio of HRs (rHR).^3^ The rHR is defined as the ratio of the HR of PFS to the HR of OS. The summary rHR across the studies was obtained by pooling the individual rHRs of each study using a random-effects model to account for the heterogeneous group of patient populations. An rHR of less than 1 would indicate that the treatment effects of PD-1 inhibitors were larger for PFS than OS, while an rHR greater than 1 would indicate that the treatment effects were larger for OS than PFS. A treatment with an rHR of less than 1 improves (benefits) PFS more than it improves OS, while a treatment with an rHR greater than 1 improves OS more than it improves PFS.

Heterogeneity among studies was assessed using the Cochrane *Q* statistic (assumption of homogeneity was considered invalid for values of *P* < .10) and quantified using an *I^2^* test. Subgroup analyses were prespecified and included drug (nivolumab or pembrolizumab), tumor type, and line of use (first line vs others). Two-sided *P* < .05 was the threshold for statistical significance.

## Results

Our search revealed 1825 potentially relevant reports, of which 12 trials^8,9,10,11,12,13,14,15,16,17,18,19,20,21^ fulfilled our eligibility criteria (eFigure 1 in the Supplement). Of these 12, 2 tested nivolumab or pembrolizumab after treatment with ipilimumab, and because of the potential for residual effects of ipilimumab, these 2 trials were not considered for primary analysis but were included as a secondary analysis.

Table 1 presents the basic characteristics of our main sample, which included 6 trials of nivolumab^8,9,10,11,12,13^ and 4 trials of pembrolizumab.^14,15,16,17^ One RCT was a phase 2 trial (Keynote 021)^17^ and the rest were phase 3. Four studies were conducted with the immunotherapy as first-line treatment and 6 with the immunotherapy as second-line treatment after chemotherapy or targeted therapy. Nine studies tested a PD-1 inhibitor as a single agent, while the Keynote 021 study tested pembrolizumab plus chemotherapy vs chemotherapy alone.^17^ Non–small cell lung cancer was the most common tumor type studied (6 RCTs [50% of the cohort]). There was 1 RCT each in melanoma, head and neck cancer, renal cell cancer, and urothelial cancer.

**Table 1.** Characteristics of Trials Included in the Meta-analysis

Source	Setting	Primary End Point	Jadad Score	Blinded	Participants, No.	PD-1 Group Treatment Protocol	Control Group Treatment Protocol	Participants in PD-1 Group, No.	Participants in Control Group, No.
Ferris et al,^8^ 2016 (Checkmate 141)	Recurrent head and neck	OS	2	No	361	Nivolumab, 3 mg/kg every 2 wk	Single-agent chemotherapy of investigator’s choice	240	121
Borghaei et al,^9^ 2015 (Checkmate 057)	Second line, nonsquamous NSCLC	OS	2	No	582	Nivolumab, 3 mg/kg every 2 wk	Docetaxel	292	290
Brahmer et al,^10^ 2015 (Checkmate 017)	Second line, squamous NSCLC	OS	2	No	272	Nivolumab, 3 mg/kg every 2 wk	Docetaxel	135	137
Robert et al,^11^ 2015 (Checkmate 066)	First line, melanoma	OS	4	Yes	418	Nivolumab, 3 mg/kg every 2 wk	Dacarbazine	210	208
Motzer et al,^12^ 2015 (Checkmate 025)	Second line, RCC	OS	3	No	821	Nivolumab, 3 mg/kg every 2 wk	Everolimus	410	411
Carbone et al,^13^ 2017 (Checkmate 026)	First line, NSCLC	PFS	2	No	541	Nivolumab, 3 mg/kg every 2 wk	Platinum-based chemotherapy	271	270
Bellmunt et al,^14^ 2017 (Keynote 045)	Second line, urothelial	OS and PFS	2	No	542	Pembrolizumab, 200 mg every 3 wk	Single-agent chemotherapy: paclitaxel, docetaxel, or vinflunine	270	272
Reck et al,^15^ 2016 (Keynote 024)	First line, NSCLC	PFS	2	No	305	Pembrolizumab, 200 mg every 3 wk	Platinum-based chemotherapy	154	151
Herbst et al,^16^ 2016 (Keynote 010)^a^	Second line, NSCLC	OS and PFS	3	No	688	Pembrolizumab, 2 mg/kg or 10 mg/kg every 3 wk	Docetaxel	345	343
Langer et al,^17^ 2016 (Keynote 021)^b^^,^^c^	First line, NSCLC	ORR	3	No	123	Pembrolizumab, 200 mg every 3 wk, plus carboplatin-pemetrexed chemotherapy with pembrolizumab plus pemetrexed maintenance	Carboplatin-pemetrexed chemotherapy with pemetrexed maintenance	60	63
**After Treatment With Ipilimumab **									
Ribas et al,^18^ 2015 and Hamid et al,^19^ 2016 (Keynote 002)^a^^,^^b^	Second line, melanoma	PFS	3	No	359	Pembrolizumab, 2 mg/kg or 10 mg/kg	Chemotherapy of investigator’s choice	180	179
Weber et al,^20^ 2015 and Larkin et al,^21^ 2018 (Checkmate 037)	Second line, melanoma	OS and ORR	2	No	405	Nivolumab, 3 mg/kg every 2 wk	Chemotherapy of investigator’s choice	272	133

Abbreviations: NSCLC, non–small cell lung cancer; ORR, overall response rate; OS, overall survival; PD-1, programmed cell death 1; PFS, progression-free survival; RCC, renal cell carcinoma.

^a^ Only the 2-mg/kg cohort of pembrolizumab has been included in this analysis.

^b^ These are the only phase 2 trials in this analysis. All other trials are phase 3.

^c^ This is the only trial where a PD-1 inhibitor was not tested as a single agent, but as a combination with chemotherapy.

Nivolumab was tested at a dose of 3 mg/kg every 2 weeks in each trial. Pembrolizumab was tested at either 2 mg/kg or 10 mg/kg or a fixed dose of 200 mg every 3 weeks. Keynote 010 and Keynote 002 trials used 2 different pembrolizumab groups of 2 mg/kg and 10 mg/kg. However, because the current FDA-approved dose of 200 mg is closer to 2 mg/kg than 10 mg/kg, we used data for the 2-mg/kg group in our study.

Traditional RECIST 1.1 criteria were used to define progression in all the included trials. The primary end points were OS in 5 RCTs (all involving nivolumab), PFS in 2 RCTs (1 nivolumab and 1 pembrolizumab), and both OS and PFS as coprimary end points in 2 RCTs of pembrolizumab (Table 1). The 1 phase 2 pembrolizumab trial, Keynote 021, used a primary end point of objective response rate.^17^ Because this trial was the only phase 2 trial, the only one with response rate as an end point, and the only trial in which a PD-1 inhibitor was not tested as a single agent, we performed all rHR analyses with and without including this trial.

### Study Quality

Most of the studies in our sample had Jadad scores of only 2 or 3 (Table 1). Only 1 study was double blind (Checkmate 066 [Jadad score 4]), while the others were open label. Trials with low Jadad scores often did not describe the methods of randomization adequately. All the studies that had PFS or response rate as their primary or coprimary end points, which require some measure of subjective judgment to assess tumor growth, were open label. All the studies reported data on median PFS and HRs of PFS and OS; however, the median OS was not yet reached for 3 trials (Table 2).

**Table 2.** Efficacy Data

		PFS				OS			
Source	Setting	PD-1 Group, mo	Control Group, mo	Difference, mo	Hazard Ratio (95% CI)	PD-1 Group, mo	Control Group, mo	Difference, mo	Hazard Ratio (CI)
Ferris et al,^8^ 2016 (Checkmate 141)	Recurrent head and neck	2.0	2.3	−0.3	0.89 (0.70-1.13)	7.5	5.1	2.4	0.70 (97.73% CI, 0.51-0.96)
Borghaei et al,^9^ 2015 (Checkmate 057)	Second line, nonsquamous NSCLC	2.3	4.2	−1.9	0.92 (0.77-1.1)	12.2	9.4	2.8	0.73 (96.00% CI, 0.59-0.89)
Brahmer et al,^10^ 2015 (Checkmate 017)	Second line, squamous NSCLC	3.5	2.8	0.7	0.62 (0.47-0.81)	9.2	6	3.2	0.59 (95.00% CI, 0.44-0.79)
Robert et al,^11^ 2015 (Checkmate 066)	First line, melanoma	5.1	2.2	2.9	0.43 (0.34-0.56)	NR	10.8	NR	0.42 (99.79% CI, 0.25-0.73)
Motzer et al,^12^ 2015 (Checkmate 025)	Second line, RCC	4.6	4.4	0.2	0.88 (0.75-1.03)	25	19.6	5.4	0.73 (98.50% CI, 0.57-0.93)
Carbone et al,^13^ 2017 (Checkmate 026)	First line, NSCLC	4.2	5.9	−1.7	1.15 (0.91-1.45)	14.4	13.2	1.2	1.02 (95.00% CI, 0.80-1.30)
Bellmunt et al,^14^ 2017 (Keynote 045)	Second line, urothelial	2.1	3.3	−1.2	0.98 (0.81-1.19)	10.3	7.4	2.9	0.73 (95.00% CI, 0.59-0.91)
Reck et al,^15^ 2016 (Keynote 024)	First line, NSCLC	10.3	6.0	4.3	0.5 (0.37-0.68)	NR	NR	NR	0.60 (95.00% CI, 0.41-0.89)
Herbst et al,^16^ 2016 (Keynote 010)^a^	Second line, NSCLC	3.9	4.0	−0.1	0.88 (0.74-1.05)	10.4	8.5	1.9	0.71 (95.00% CI, 0.58-0.88)
Langer et al,^17^ 2016 (Keynote 021)^b^^,^^c^	First line, NSCLC	13.0	8.9	4.1	0.53 (0.31-0.91)	NR	NR	NR	0.90 (95.00% CI, 0.42-1.91)
**After Treatment With Ipilimumab **									
Ribas et al,^18^ 2015 and Hamid et al,^19^ 2016 (Keynote 002)^a^^,^^b^	Second line, melanoma	2.9	2.7	0.2	0.57 (0.45-0.73)	13.4	11.0	2.4	0.86 (95.00% CI, 0.67-1.10)
Weber et al,^20^ 2015 and Larkin et al,^21^ 2018 (Checkmate 037)	Second line, melanoma	3.1	3.7	−0.6	1.0 (0.78-1.44)	15.7	14.4	1.3	0.95 (95.54% CI, 0.73-1.24)

Abbreviations: NR, not reached; NSCLC, non–small cell lung cancer; OS, overall survival; PD-1, programmed cell death 1; PFS, progression-free survival; RCC: renal cell carcinoma.

^a^ Only the 2-mg/kg cohort of pembrolizumab has been included in this analysis.

^b^ These are the only phase 2 trials in this analysis. All other trials are phase 3.

^c^ This is the only trial in which a PD-1 inhibitor was not tested as a single agent, but as a combination with chemotherapy.

### Study Participants

In the 10 RCTs included in the primary meta-analysis, 4653 participants were randomized (PD-1 cohort: 2387; control: 2266). The majority of participants (2995 [64%]) were from nivolumab trials. The secondary analysis included 5417 participants from 12 RCTs (PD-1 cohort: 2839; control cohort: 2578). All trials restricted enrollment to patients with an Eastern Cooperative Oncology Group performance status score of 0 or 1 (on a scale of 0-5, with 0 indicating a patient is fully active and able to carry on activity without restriction and 5 indicating death), except for Keynote 045,^14^ which enrolled patients with a performance status of up to 2, and Checkmate 025,^12^ which enrolled patients with Karnofsky performance status score of 70 or above (on a scale of 0-100, with 0 indicating death and 100 indicating patient has normal activity with no symptoms).

### Correlation Between PFS and OS

Data for median PFS were available for all studies. Data for median OS were not available for 3 studies (Table 2). Thus, the correlation between PFS and OS could only be obtained from 7 RCTs. The correlation between median OS and median PFS was not significant (n = 7; *r* = 0.676; *R^2^* = 0.457; *P* = .09) (eFigure 2 in the Supplement). The gain in OS also did not correlate with the gain in PFS (n = 7; *r* = 0.474; *R^2^* = 0.225; *P* = .28) (eFigure 3 in the Supplement). However, there was significant correlation between the HRs of PFS and OS (n = 10; *r* = 0.637; *R^2^* = 0.406; *P* = .048) (eFigure 4 in the Supplement).

### Difference in Treatment Effect Sizes Between PFS and OS

All the trials in our sample reported HRs for both PFS and OS. The HRs for OS were statistically significant for 8 trials (80%), whereas the HRs for PFS were statistically significant in only 4 trials (40%) (Table 2). In 5 trials (50%), there was benefit seen for OS without any PFS benefit. In only 1 trial, PFS benefit occurred without any benefit in OS. This was the Keynote 021 trial.^17^

Among the 10 trials in the primary analysis, the treatment effect sizes were greater for OS than for PFS (pooled rHR, 1.18; 95% CI, 1.06-1.31; *P* = .002) (Figure). Because all the HRs for OS and the HRs for PFS in the individual trials were on the same side of null, a pooled rHR of 1.18 means the PD-1 inhibitors have a protective effect (improvement) on OS and that the HRs for PFS on average were 18% more than the HRs for OS. The included studies were statistically nonheterogeneous (*Q* = 6.24; *P* = .72; *I^2^* = 0%). When excluding the Keynote 021 study, the rHR was 1.19 (95% CI, 1.07-1.32; *P* = .001).

**Figure.**
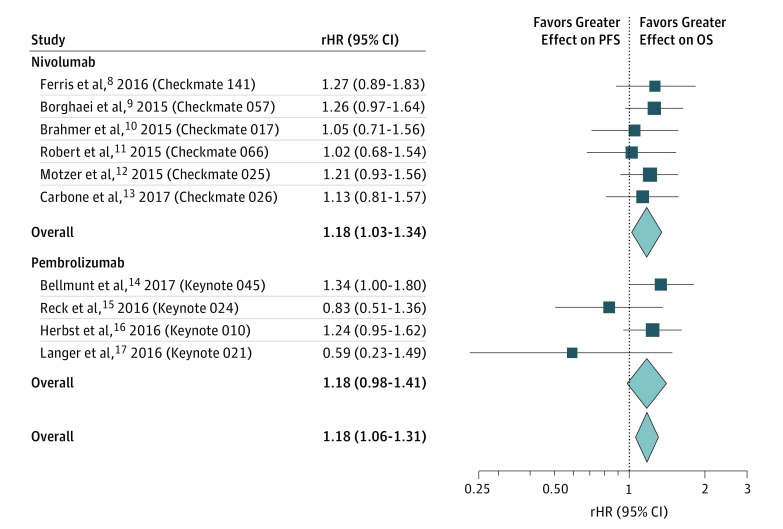
Pooling of Ratio of Hazard Ratios (rHR) of Progression-Free Survival (PFS) and Overall Survival (OS) Among the Randomized Clinical Trials of Programmed Cell Death 1 (PD-1) Inhibitors Among the 10 trials in the primary analysis, the treatment effect sizes were greater for OS than for PFS.

### Subgroup Analyses

The treatment effect sizes were greater for OS than for PFS for both nivolumab and pembrolizumab, but missed statistical significance for pembrolizumab (nivolumab rHR, 1.18; 95% CI, 1.03-1.34; *P* = .01 vs pembrolizumab rHR, 1.18; 95% CI, 0.98-1.41; *P* = .07). There was no heterogeneity among the nivolumab studies (*I^2^* = 0%) but some heterogeneity among the pembrolizumab studies (*I^2^* = 39.6%). However, when the outlier pembrolizumab trial was excluded, the effect size on OS was greater than for PFS for pembrolizumab (rHR, 1.21; 95% CI, 1.01-1.45).

The treatment effect sizes between PFS and OS for RCTs of non–small cell lung cancer trials were similar (rHR, 1.14; 95% CI, 0.99-1.31), whereas for the other cancer types, there was a greater effect on OS than on PFS (rHR, 1.23; 95% CI, 1.05-1.44). The observed treatment effect was also greater on OS than on PFS for trials conducted using the drug as a second-line or later treatment (rHR, 1.24; 95% CI, 1.10-1.40), but not for those trials conducted in the first-line setting (rHR, 0.99; 95% CI, 0.79-1.24) (Table 3).

**Table 3.** Subgroup Analysis

Subgroup	Studies, No.	Summary rHR (95% CI)	*P* Value	Heterogeneity Test		
				*Q*	*P* Value	*I^2^,* %
Drug type						
Nivolumab	6	1.177 (1.033-1.341)	.01	1.29	.94	0
Pembrolizumab	4	1.178 (0.984-1.411)	.07	4.97	.17	39.6
Overall	10	1.178 (1.060-1.309)	.002	6.25	.72	0
Tumor type						
Non–small cell lung cancer	6	1.138 (0.986-1.313)	.08	4.60	.46	0
Other tumor types	4	1.227 (1.049-1.435)	.01	1.17	.76	0
Overall	10	1.178 (1.060-1.309)	.002	6.25	.72	0
Line of therapy						
First line	4	0.992 (0.794-1.240)	.95	2.29	.52	0
Second or later lines	6	1.238 (1.098-1.396)	<.001	1.03	.96	0
Overall	10	1.178 (1.060-1.309)	.002	6.25	.72	0

Abbreviation: rHR, ratio of hazard ratios.

### Secondary Analyses

The 2 RCTs that were conducted in patients who had already been treated with ipilimumab were Keynote 002, which was a phase 2 trial of pembrolizumab,^18,19^ and Checkmate 037,^20^ which was a phase 3 trial of nivolumab. Both were conducted in advanced melanoma. As secondary analysis, we also tested the rHR by including these 2 RCTs and found that the effect sizes for PFS and OS were similar for the combined analysis of these 2 trials as well as in the overall population when they were included (eTable in the Supplement).

## Discussion

In this study, which to our knowledge is the first to evaluate the correlation and differences in treatment effect sizes between PFS and OS in PD-1 inhibitor trials, we found that OS was poorly correlated with PFS with respect to both medians and absolute gains. However, unlike with targeted agents or chemotherapies, this was not because improvement in PFS benefit did not translate to OS. Rather, OS benefits were observed without any apparent benefit in PFS. Indeed, the HRs of PFS were on average greater than the HRs of OS by as much as 18%. Also, the correlation between PFS and OS in terms of HRs was significant.

The lack of correlation between the medians and absolute difference in medians occurring with a significant correlation between the HRs of OS and PFS is not surprising for 2 reasons. First, the correlation between the medians was based on only 7 trials because the median OS was unavailable for 3 RCTs, but the HR correlation was calculated for 10 trials, increasing the power. Second, medians are not a good marker of efficacy for immuno-oncology trials, and the HR should capture the benefit better than the median.^22,23^ The correlation between PFS and OS will get clearer as more trials are published and a larger sample can be analyzed.

Several hypotheses might explain our findings of greater benefits in OS than PFS with PD-1 inhibitors. First, PFS in all these trials was defined using the traditional RECIST criteria, which were developed in the era before immunotherapy. It has been reported that traditional RECIST criteria fail to properly capture the concept of disease progression with immunotherapies that have atypical response patterns.^6^ Although immunotherapy-specific RECIST criteria have been proposed, they have not been used in trials yet.^24^ Failure of traditional RECIST criteria to define PFS of immunotherapies might be 1 reason for smaller benefits in PFS vs OS with the trials of PD-1 inhibitors.^25^

Second, because PD-1 inhibitors have residual efficacy for a longer duration, these drugs could affect OS more than PFS even after the discontinuation of treatment.^4,5^ Some patients experience a very durable response with immunotherapies, and thus the benefit in OS seen in the overall population could primarily be driven by extraordinary benefit in a select few patients. However, the tail effect that has been widely reported with ipilimumab has yet to be observed with PD-1 inhibitors.^26^

A third explanation for our findings might be pseudoprogression,^27^ in which the tumor first grows in size due to T-cell infiltrate before undergoing shrinkage. This phenomenon might lead the investigators to consider the response progressive disease under RECIST criteria when, in fact, the patient could respond later, ultimately leading to benefit in OS.

The subgroup analyses in our study suggested that the effect on OS vs PFS was greater for nivolumab than pembrolizumab, greater for tumor types other than non–small cell lung cancer, and greater when used in second or later lines vs first line of therapy. This is in keeping with the past observations that the crossing-over of PFS curves has been seen in nivolumab trials but not pembrolizumab, and that the phenomenon of pseudoprogression is not as common in non–small cell lung cancer as in other tumor types.^28^ However, these analyses should be considered hypothesis generating, rather than confirmatory, because of the small sample sizes and the lack of heterogeneity among the trials.

### Limitations

This study has limitations. First, it involves only RCTs with PD-1 inhibitors and is not generalizable to other checkpoint inhibitors. Second, when 2 studies conducted in patients previously treated with ipilimumab were included, the difference in treatment effect sizes between PFS and OS was no longer statistically significant. Third, most studies included in this analysis used PD-1 inhibitors as a single agent; the result when these drugs are used in combination remains unknown. Fourth, medians are not always considered an appropriate metric for assessing correlation between PFS and OS with immunotherapy drugs, and alternative metrics have been proposed but not yet adopted in trials.^22,23^ Furthermore, the proportion of patients responding to and receiving benefit from PD-1 inhibitors differs by tumor type and, thus, this trial-level analysis may not hold true for the individual patient. Another limitation inherent in all RCTs of immunotherapy drugs is the use of immunotherapies after progression, which can confound OS. These data were available for 5 studies, of which 4 reported significant benefit in OS. Thus, the receipt of immunotherapies after progression does not seem to affect this analysis.

## Conclusions

Most previous analyses of the correlation between OS and PFS have focused on tumor types.^2^ In the case of immunotherapies, the correlation between OS and surrogate outcomes might reasonably be considered a function of the drug class rather than the tumor biology. For PD-1 inhibitors, we found that OS is not correlated with PFS measured by traditional RECIST criteria; however, the HRs were correlated. By contrast, PD-1 inhibitors may have larger effects on OS than on PFS, which would be unprecedented in oncology therapeutics. These results support the rationale of using OS as the primary end point of future phase 3 trials of PD-1 inhibitors and discourage the use of PFS as a sole primary end point, as the latter may provide misleading information about the efficacy of these drugs.

## Notes

Supplement. **eTable.** Primary and Secondary Analysis Excluding and Including the 2 RCTs That Tested PD-1 Inhibitors in the Post-ipilimumab Setting **eFigure 1.** Selection of Randomized Controlled Trials (RCTs) Included in the Analysis **eFigure 2.** Correlation Between Median Overall Survival (OS) and Median Progression-Free Survival (PFS) in the RCTs of PD-1 Inhibitors **eFigure 3.** Correlation Between Gain in Median Overall Survival and Gain in Median Progression-Free Survival in the RCTs of PD-1 Inhibitors **eFigure 4.** Correlation Between Hazard Ratio (HR) of OS and HR of PFS in the RCTs of PD-1 Inhibitors

## References

[1] Kemp R, Prasad V Surrogate endpoints in oncology: when are they acceptable for regulatory and clinical decisions, and are they currently overused? BMC Med. 2017;15(1):. 28728605 10.1186/s12916-017-0902-9 PMC5520356

[2] Prasad V, Kim C, Burotto M, Vandross A The strength of association between surrogate end points and survival in oncology: a systematic review of trial-level meta-analyses. JAMA Intern Med. 2015;175(8):1389-. 26098871 10.1001/jamainternmed.2015.2829

[3] Tan A, Porcher R, Crequit P, Ravaud P, Dechartres A Differences in treatment effect size between overall survival and progression-free survival in immunotherapy trials: a meta-epidemiologic study of trials with results posted at ClinicalTrials.gov. J Clin Oncol. 2017;35(15):1686-1694. 28375786 10.1200/JCO.2016.71.2109

[4] Lipson EJ, Sharfman WH, Drake CG, Durable cancer regression off-treatment and effective reinduction therapy with an anti-PD-1 antibody. Clin Cancer Res. 2013;19(2):462-468. 23169436 10.1158/1078-0432.CCR-12-2625 PMC3548952

[5] Queirolo P, Spagnolo F Atypical responses in patients with advanced melanoma, lung cancer, renal-cell carcinoma and other solid tumors treated with anti-PD-1 drugs: a systematic review. Cancer Treat Rev. 2017;59:71-78. 28756306 10.1016/j.ctrv.2017.07.002

[6] Gyawali B, Ota A, Ando Y Nivolumab in nonsquamous non-small-cell lung cancer. N Engl J Med. 2016;374(5):492-494. 26840146 10.1056/NEJMc1514790

[7] Jadad AR, Moore RA, Carroll D, Assessing the quality of reports of randomized clinical trials: is blinding necessary? Control Clin Trials. 1996;17(1):1-12. 8721797 10.1016/0197-2456(95)00134-4

[8] Ferris RL, Blumenschein G Jr, Fayette J, Nivolumab for recurrent squamous-cell carcinoma of the head and neck. N Engl J Med. 2016;375(19):1856-1867. 27718784 10.1056/NEJMoa1602252 PMC5564292

[9] Borghaei H, Paz-Ares L, Horn L, Nivolumab versus docetaxel in advanced nonsquamous non-small-cell lung cancer. N Engl J Med. 2015;373(17):1627-1639. 26412456 10.1056/NEJMoa1507643 PMC5705936

[10] Brahmer J, Reckamp KL, Baas P, Nivolumab versus docetaxel in advanced squamous-cell non-small-cell lung cancer. N Engl J Med. 2015;373(2):123-135. 26028407 10.1056/NEJMoa1504627 PMC4681400

[11] Robert C, Long GV, Brady B, Nivolumab in previously untreated melanoma without *BRAF* mutation. N Engl J Med. 2015;372(4):320-330. 25399552 10.1056/NEJMoa1412082

[12] Motzer RJ, Escudier B, McDermott DF, ; CheckMate 025 Investigators Nivolumab versus everolimus in advanced renal-cell carcinoma. N Engl J Med. 2015;373(19):1803-1813. 26406148 10.1056/NEJMoa1510665 PMC5719487

[13] Carbone DP, Reck M, Paz-Ares L, ; CheckMate 026 Investigators First-line nivolumab in stage IV or recurrent non-small-cell lung cancer. N Engl J Med. 2017;376(25):2415-2426. 28636851 10.1056/NEJMoa1613493 PMC6487310

[14] Bellmunt J, de Wit R, Vaughn DJ, ; KEYNOTE-045 Investigators Pembrolizumab as second-line therapy for advanced urothelial carcinoma. N Engl J Med. 2017;376(11):1015-1026. 28212060 10.1056/NEJMoa1613683 PMC5635424

[15] Reck M, Rodríguez-Abreu D, Robinson AG, ; KEYNOTE-024 Investigators Pembrolizumab versus chemotherapy for PD-L1-positive non-small-cell lung cancer. N Engl J Med. 2016;375(19):1823-1833. 27718847 10.1056/NEJMoa1606774

[16] Herbst RS, Baas P, Kim DW, Pembrolizumab versus docetaxel for previously treated, PD-L1-positive, advanced non-small-cell lung cancer (KEYNOTE-010): a randomised controlled trial. Lancet. 2016;387(10027):1540-1550. 26712084 10.1016/S0140-6736(15)01281-7

[17] Langer CJ, Gadgeel SM, Borghaei H, ; KEYNOTE-021 investigators Carboplatin and pemetrexed with or without pembrolizumab for advanced, non-squamous non-small-cell lung cancer: a randomised, phase 2 cohort of the open-label KEYNOTE-021 study. Lancet Oncol. 2016;17(11):1497-1508. 27745820 10.1016/S1470-2045(16)30498-3 PMC6886237

[18] Ribas A, Puzanov I, Dummer R, Pembrolizumab versus investigator-choice chemotherapy for ipilimumab-refractory melanoma (KEYNOTE-002): a randomised, controlled, phase 2 trial. Lancet Oncol. 2015;16(8):908-918. 26115796 10.1016/S1470-2045(15)00083-2 PMC9004487

[19] Hamid O, Puzanov I, Dummer R, Final overall survival for KEYNOTE-002: pembrolizumab (pembro) versus investigator-choice chemotherapy (chemo) for ipilimumab (ipi)-refractory melanoma. Ann Oncol. 2016;27(suppl 6):1107O.26940689

[20] Weber JS, D’Angelo SP, Minor D, Nivolumab versus chemotherapy in patients with advanced melanoma who progressed after anti-CTLA-4 treatment (CheckMate 037): a randomised, controlled, open-label, phase 3 trial. Lancet Oncol. 2015;16(4):375-384. 25795410 10.1016/S1470-2045(15)70076-8

[21] Larkin J, Minor D, D’Angelo S, Overall survival in patients with advanced melanoma who received nivolumab versus investigator’s choice chemotherapy in CheckMate 037: a randomized, controlled, open-label phase III trial. J Clin Oncol. 2018;36(4):383-390. 28671856 10.1200/JCO.2016.71.8023 PMC6804912

[22] Pak K, Uno H, Kim DH, Interpretability of cancer clinical trial results using restricted mean survival time as an alternative to the hazard ratio. JAMA Oncol. 2017;3(12):1692-1696. 28975263 10.1001/jamaoncol.2017.2797 PMC5824272

[23] Hoering A, Durie B, Wang H, Crowley J End points and statistical considerations in immuno-oncology trials: impact on multiple myeloma. Future Oncol. 2017;13(13):1181-1193. 28395525 10.2217/fon-2016-0504 PMC5705823

[24] Seymour L, Bogaerts J, Perrone A, ; RECIST Working Group iRECIST: guidelines for response criteria for use in trials testing immunotherapeutics. Lancet Oncol. 2017;18(3):e143-e152. 28271869 10.1016/S1470-2045(17)30074-8 PMC5648544

[25] Hodi FS, Hwu W-J, Kefford R, Evaluation of immune-related response criteria and RECIST v1.1 in patients with advanced melanoma treated with pembrolizumab. J Clin Oncol. 2016;34(13):1510-1517. 26951310 10.1200/JCO.2015.64.0391 PMC5070547

[26] Schadendorf D, Hodi FS, Robert C, Pooled analysis of long-term survival data from phase II and phase III trials of ipilimumab in unresectable or metastatic melanoma. J Clin Oncol. 2015;33(17):1889-1894. 25667295 10.1200/JCO.2014.56.2736 PMC5089162

[27] Chiou VL, Burotto M Pseudoprogression and immune-related response in solid tumors. J Clin Oncol. 2015;33(31):3541-3543. 26261262 10.1200/JCO.2015.61.6870 PMC4622096

[28] Nishino M, Ramaiya NH, Chambers ES, Immune-related response assessment during PD-1 inhibitor therapy in advanced non-small-cell lung cancer patients. J Immunother Cancer. 2016;4:84. 28018599 10.1186/s40425-016-0193-2 PMC5168591

