# Supplementary material for: A Comparison of Response Patterns for Progression-Free Survival and Overall Survival Following Treatment for Cancer With PD-1 Inhibitors: A Meta-analysis of Correlation and Differences in Effect Sizes
Source: JAMA Netw Open. 2018 Jun 22;1(2):e180416. doi: 10.1001/jamanetworkopen.2018.0416 (PMC6324401; doi:10.1001/jamanetworkopen.2018.0416)
Supplement: Supplement. — eTable. Primary and Secondary Analysis Excluding and Including the 2 RCTs That Tested PD-1 Inhibitors in the Post-ipilimumab Setting eFigure 1. Selection of Randomized Controlled Trials (RCTs) Included in the Analysis eFigure 2. Correlation Between Median Overall Survival (OS) and Median Progression-Free Survival (PFS) in the RCTs of PD-1 Inhibitors eFigure 3. Correlation Between Gain in Median Overall Survival and Gain in Median Progression-Free Survival in the RCTs of PD-1 Inhibitors eFigure 4. Correlation Between Hazard Ratio (HR) of OS and HR of PFS in the RCTs of PD-1 Inhibitors [file jamanetwopen-1-e180416-s001.pdf]

Supplementary Online Content

Gyawali B, Hey SP, Kesselheim AS. A comparison of response patterns for progression-free survival and overall survival following treatment for cancer with PD-1 inhibitors: a meta-analysis of correlation and differences in effect sizes. *JAMA Netw Open*. 2018;1(2):e180416. doi:10.1001/jamanetworkopen.2018.0416

eTable. Primary and Secondary Analysis Excluding and Including the 2 RCTs That Tested PD-1 Inhibitors in the Post-ipilimumab Setting

eFigure 1. Selection of Randomized Controlled Trials (RCTs) Included in the Analysis

eFigure 2. Correlation Between Median Overall Survival (OS) and Median Progression-Free Survival (PFS) in the RCTs of PD-1 Inhibitors

eFigure 3. Correlation Between Gain in Median Overall Survival and Gain in Median Progression-Free Survival in the RCTs of PD-1 Inhibitors

eFigure 4. Correlation Between Hazard Ratio (HR) of OS and HR of PFS in the RCTs of PD-1 Inhibitors

This supplementary material has been provided by the authors to give readers additional information about their work.

© 2018 Gyawali B et al. *JAMA Network Open.*

eTable 1: Secondary analyses including trials from post-ipilimumab setting

Setting	N of studies	Summary rHR	95% CI	P-value	Heterogeneity test
Immune checkpoint inhibitors naïve	10	1.178	1.060-1.309	0.002	Q=6.25, P = 0.72, I^2 = 0.0
Post-ipilimumab	2	0.833	0.652-1.065	0.146	Q =3.15, P = 0.08, I^2 = 68.25
Overall	12	1.099	0.975-1.239	0.123	Q= 15.82, P =0.148, I^2=30.47

© 2018 Gyawali B et al. *JAMA Network Open.*

eFigure 1: Selection of Randomized Controlled Trials (RCTs) included in the analysis.

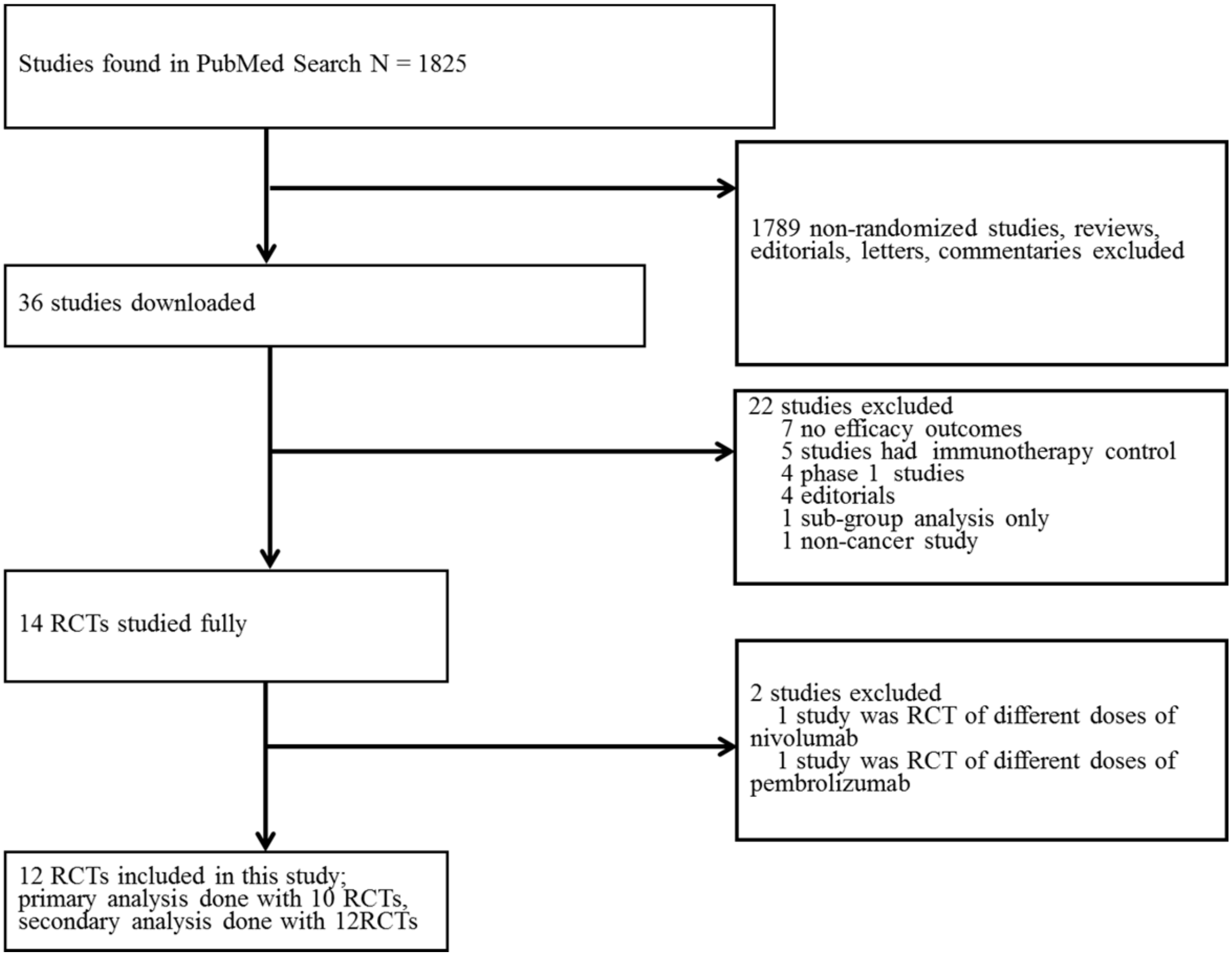

© 2018 Gyawali B et al. *JAMA Network Open.*

***eFigure 2*: Correlation between median overall survival (OS) and median progression-free survival (PFS) in the RCTs of PD-1 inhibitors.**

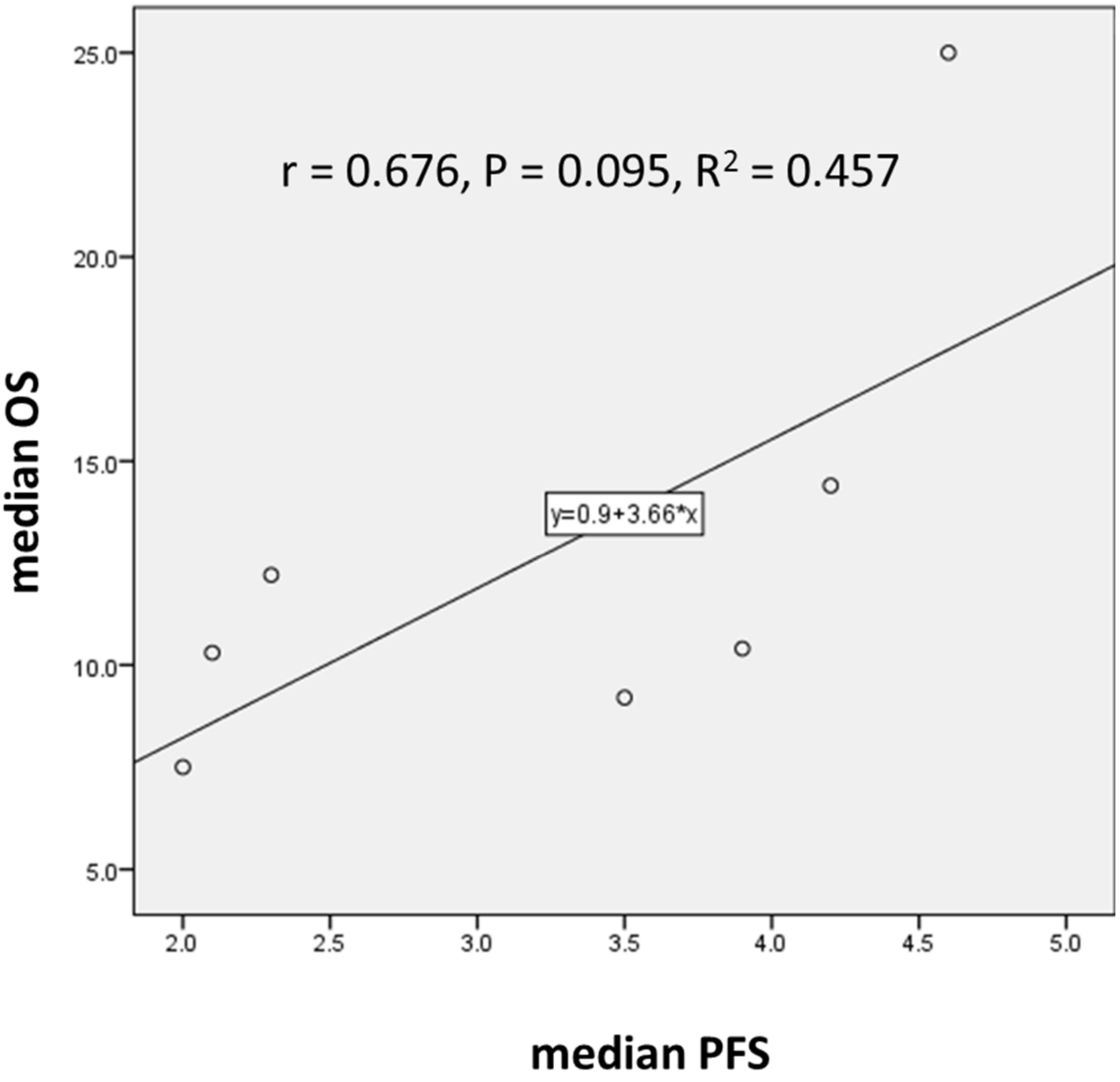

© 2018 Gyawali B et al. *JAMA Network Open.*

***eFigure 3*: Correlation between gain in median overall survival (∆OS) and gain in median progression-free survival (∆PFS) in the RCTs of PD-1 inhibitors**

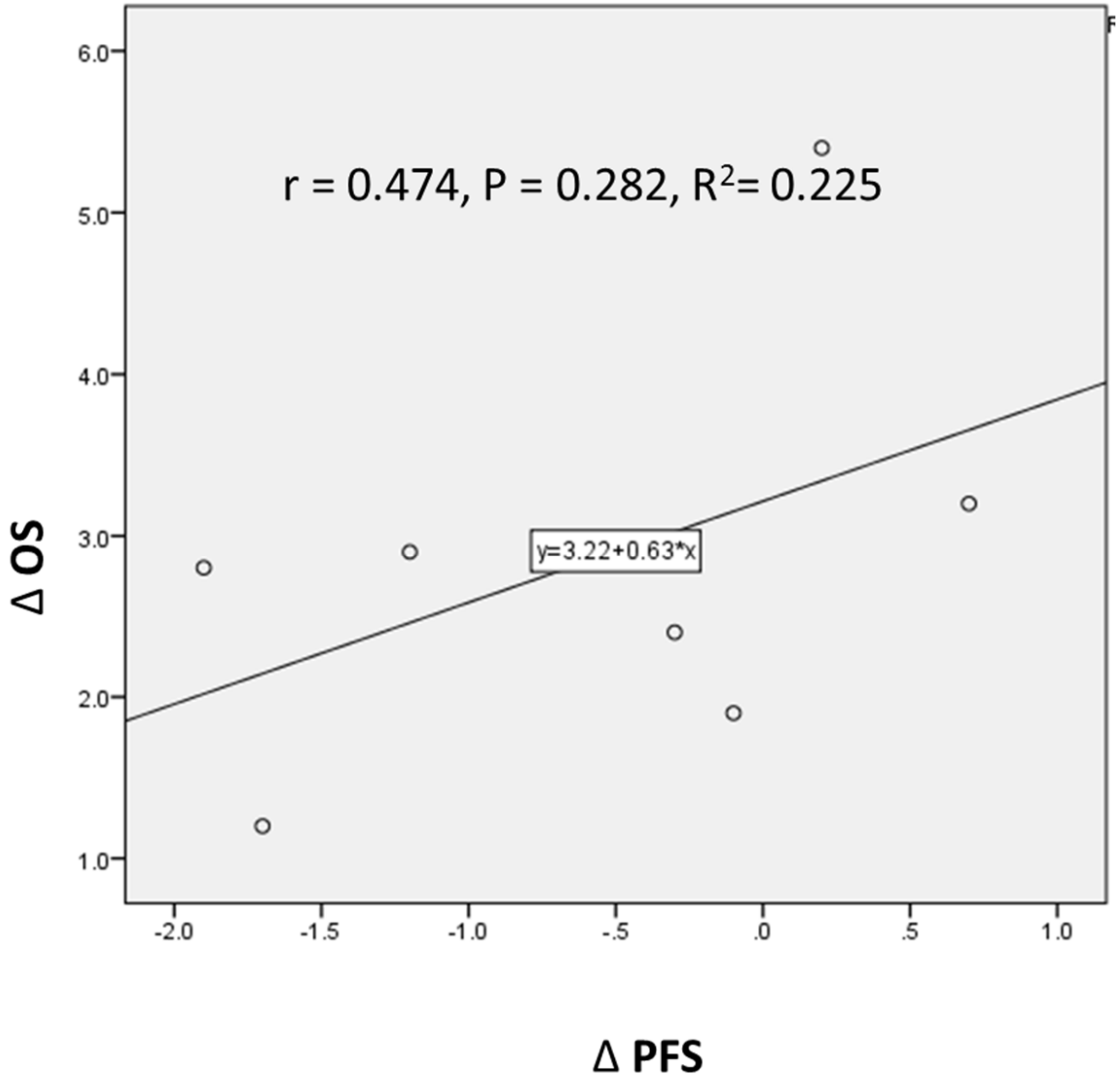

© 2018 Gyawali B et al. *JAMA Network Open.*

***eFigure 4:* Correlation between hazard ratio (HR) of OS and HR of PFS in the RCTs of PD-1 inhibitors**

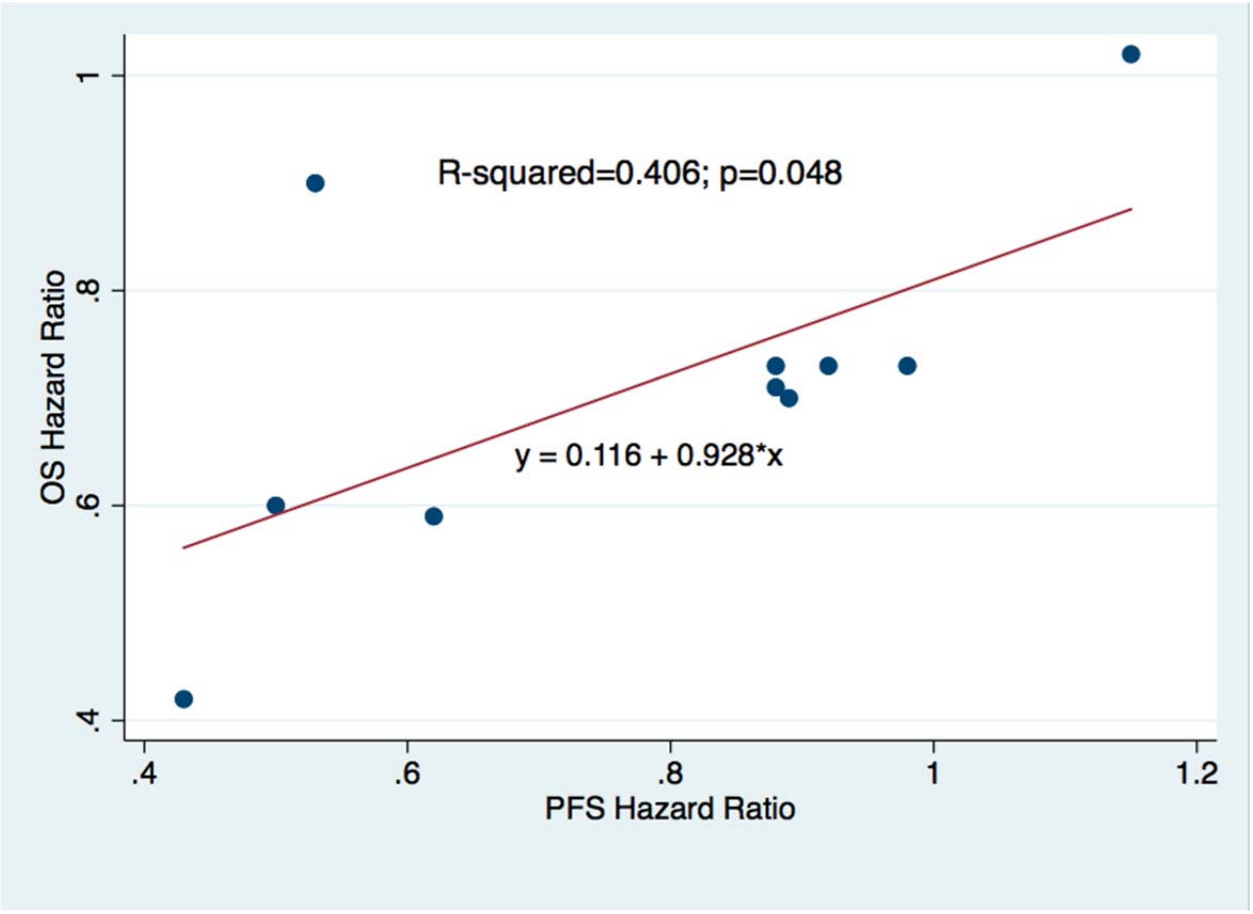

© 2018 Gyawali B et al. *JAMA Network Open.*
